# Development and validation of a risk prediction model for postpartum urinary incontinence in primiparas using clinical and pelvic floor ultrasound characteristics

**DOI:** 10.3389/fmed.2025.1710133

**Published:** 2025-11-25

**Authors:** Shaofeng Guo, Zeyang Dong, Jian Zhang, Peihua Zhu, Bin Huang

**Affiliations:** 1 Department of Ultrasound, Zhejiang Hospital, Hangzhou, Zhejiang, China; 2 The Second School of Clinical Medicine, Zhejiang Chinese Medical University, Hangzhou, Zhejiang, China

**Keywords:** urinary incontinence, stress, postpartum period, pelvic floor, ultrasonography, predictive model, primipara

## Abstract

**Objective:**

To develop and validate a nomogram prediction model for postpartum stress urinary incontinence (SUI) in primiparous women, and to evaluate its clinical predictive performance.

**Methods:**

This retrospective study enrolled 447 primiparous women who delivered at Zhejiang Hospital and completed follow-up at 6–8 weeks postpartum between July 2022 and January 2024. Clinical characteristics and three-dimensional pelvic floor ultrasound parameters were collected. Participants were randomly assigned to a training set (*n* = 312) and a testing set (*n* = 135) in a 7:3 ratio. Based on the presence or absence of SUI, participants were categorized into an SUI group (*n* = 158) and a non-SUI group (*n* = 289). Independent risk factors were identified using multivariate logistic regression and incorporated into a predictive nomogram, which was subsequently validated.

**Results:**

Operative vaginal delivery, bladder neck position during maximal Valsalva, urethral rotation angle, retrovesical angle during Valsalva, and levator hiatus area were identified as independent predictors of postpartum SUI. The nomogram demonstrated excellent discriminative ability, with an area under the curve (AUC) of 0.93 (95% CI: 0.91–0.96) in both the training and testing cohorts. Calibration curves showed strong concordance between predicted and observed outcomes. Decision curve analysis further confirmed the clinical utility of the model.

**Conclusion:**

The nomogram, which integrates clinical and sonographic variables, shows promising potential as an individualized tool for predicting the risk of postpartum stress urinary incontinence in primiparous women, thereby supporting early screening and timely intervention.

## Introduction

According to the definition by the International Continence Society (ICS), stress urinary incontinence (SUI) refers to the involuntary leakage of urine from the urethra during activities that cause a sudden increase in intra-abdominal pressure, such as sneezing, coughing, or laughing (1). Primiparous pregnancy is a major risk factor for the development of SUI, with the childbirth period being a key contributor (2, 3). Epidemiological studies report that the incidence of postpartum SUI ranges from 16.41% to 25.00% (4).

The postpartum period represents a particularly critical stage for the onset and recognition of SUI. During this time, physiological changes in pelvic floor structure and function, coupled with the psychological adjustments associated with motherhood, can significantly influence how women perceive and respond to urinary symptoms. Many primiparas feel embarrassed and anxious about unpredictable urine leakage, compelling them to withdraw from routine social engagements and physical activities. This not only impedes their successful reintegration into social life but can also disrupt their postpartum recovery trajectories (5). More critically, influenced by traditional beliefs or lack of awareness, some primiparas and their families mistakenly consider postpartum stress urinary incontinence as a ‘normal consequence of childbirth’, consequently enduring the condition silently rather than proactively seeking professional help, which often delays the optimal intervention timing (6).

Current evidence suggests that the period from 6 weeks to 6 months postpartum represents a critical window for the recovery of pelvic floor function. Initiating standardised rehabilitation interventions during this stage can effectively improve pelvic floor muscle dynamics and functional outcomes (7). In this context, three-dimensional pelvic floor ultrasound has emerged as a primary imaging modality due to its accessibility, cost-effectiveness, high reproducibility, and ability to provide dynamic, spatially accurate visualization of pelvic floor structures and organ relationships (8).

In recent years, growing attention has been directed toward imaging-based evaluations of postpartum SUI. However, there remains a lack of systematic and user-friendly predictive models specifically designed for primiparous women in the early postpartum period. Therefore, the present study aims to integrate clinical risk factors with three-dimensional pelvic floor ultrasound parameters to identify independent predictors of postpartum SUI and to construct a nomogram prediction model. The goal is to provide evidence-based support for the early identification of high-risk individuals and to guide the implementation of individualized pelvic floor rehabilitation strategies in clinical practice.

## Materials and methods

### Study participants

**Figure 1:**
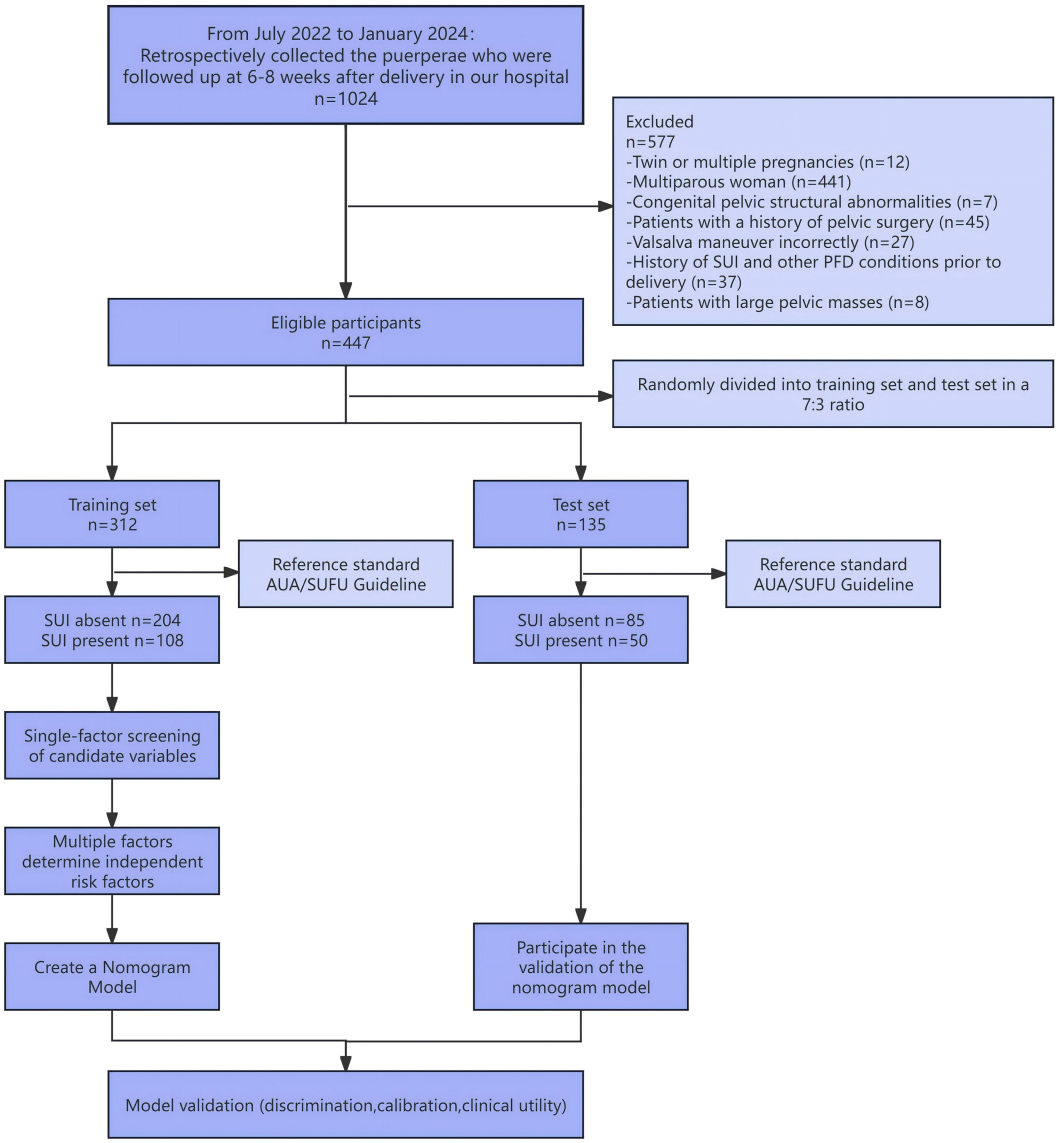
Participant flow diagram.

This study was a single-center retrospective study involving 447 primiparous women who delivered at Zhejiang Hospital between July 2022 and January 2024 and underwent postpartum follow-up and three-dimensional pelvic floor ultrasound examination at the outpatient clinic 6–8 weeks after delivery. The flow of participants through each stage of the study is presented in Figure 1. Inclusion criteria were: 1. Women who gave birth at our hospital and returned for a follow-up examination 6–8 weeks postpartum; 2. Primiparous women with a single pregnancy; 3. Women capable of performing a proper Valsalva manoeuvre; 4. Women with complete clinical records. Exclusion criteria: 1. Those with congenital pelvic structural abnormalities; 2. Those who have undergone previous pelvic-related surgeries; 3. Those with large pelvic masses; 4. A history of chronic cough or long-standing constipation. 5. Those with a history of SUI or other Pelvic Floor Disorder (PFD) conditions prior to delivery; This study has been reviewed and approved by the Zhejiang Hospital Ethics Committee, with exemption from informed consent (ethics approval number: IRB-2024-049 K).

### Study design

The diagnostic criteria for SUI were based on established clinical guidelines (9) and included the following: 1. Involuntary leakage of urine from the urethral orifice during activities that increase intra-abdominal pressure, such as sneezing, coughing, laughing, or physical exertion. The leakage resolves once the pressure subsides; 2. A positive result on either the cough stress test or the 1-h pad test.

Three-dimensional pelvic floor ultrasound examinations were performed using a GE Voluson E10 colour Doppler ultrasound system equipped with a three-dimensional volume transducer, operating at a frequency range of 4–8 MHz. Prior to the examination, patients were instructed to void their bowels and bladder at least 15 min in advance and were trained to perform an effective Valsalva manoeuvre.

During the examination, patients were placed in the lithotomy position and instructed to breathe naturally. After adjusting the machine settings, a sterile coupling agent was applied to the surface of the probe, and a disposable probe cover was used. The labia were gently separated, and the probe was positioned firmly against the perineum between the labia majora. A static mid-sagittal image of the pelvic floor was first obtained at rest, clearly visualising anatomical structures including the pubic symphysis, urethra, bladder neck, partial bladder, vagina, rectum, and anal canal (10).

Subsequently, patients were instructed to perform a maximal and effective Valsalva manoeuvre. Ultrasound images were collected during both the resting state and maximum Valsalva manoeuvre and were stored separately for further analysis. Measurements of pelvic floor parameters were performed using the built-in software of the ultrasound system, and all measurements were taken at both rest and maximal strain for each subject.

### Observation target

Clinical and obstetric data were obtained by reviewing the hospital’s electronic medical record system. The following information was collected at the time of maternal admission: age, height, weight, body mass index (BMI), smoking history, alcohol consumption history, presence of gestational hypertension [diagnosed according to the Guidelines for the Diagnosis and Treatment of Gestational Hypertensive Disorders (11)], and presence of gestational diabetes mellitus (GDM) [diagnosed based on the criteria outlined by the Diabetes Branch of the Chinese Medical Association (12)]. Additional variables included total weight gain during pregnancy, gestational age at delivery, mode of delivery, neonatal sex, neonatal birth weight, use of assisted vaginal delivery (forceps), occurrence of vaginal tears and/or episiotomy, and whether the delivery was classified as precipitous (12) or difficult (13). All data were entered into Microsoft Excel and independently cross-checked by two researchers to ensure accuracy.

Pelvic floor function was evaluated using three-dimensional pelvic floor ultrasound. The following parameters were recorded:

1) Bladder neck position at rest: The vertical distance between the bladder neck and the reference line in the resting state. Values above the reference line are recorded as positive; values below are recorded as negative.
2) Bladder neck position during maximal Valsalva manoeuvre: The vertical distance between the bladder neck and the reference line at maximal Valsalva. Positive and negative values are recorded as above.
3) Bladder neck mobility: Calculated as the difference between bladder neck positions at rest and during maximal Valsalva manoeuvre.
4) Urethral inclination angle at rest: The angle between the proximal urethra and the midline of the body in the resting state.
5) Urethral inclination angle during maximal Valsalva manoeuvre: The angle between the proximal urethra and the body’s midline during maximal Valsalva.
6) Urethral rotation angle: Defined as the difference between the urethral inclination angle at rest and during maximal Valsalva.
7) Posterior bladder angle at rest: The angle formed between the proximal urethra and the posterior wall of the bladder in the resting state.
8) Posterior bladder angle during maximal Valsalva manoeuvre: The angle between the proximal urethra and the posterior bladder wall during maximal Valsalva.
9) Levator hiatus area: The maximum cross-sectional area of the levator ani muscle hiatus during maximal Valsalva manoeuvre.

All ultrasound examinations were performed by experienced sonographers using standardized protocols. Each parameter was measured at least three times, and the mean value was used for analysis to ensure measurement reliability.

### Statistical analyses

All statistical analyses were performed using SPSS software (version 26.0, IBM Corp., Armonk, NY, USA). Categorical variables were presented as frequencies and percentages [*n* (%)] and compared using the chi-square test. Continuous variables following a normal distribution were expressed as mean ± standard deviation (SD) and analysed using the independent samples *t*-test. Non-normally distributed continuous variables were presented as median (interquartile range) [M (P25, P75)] and compared using the Mann–Whitney *U* test. A *p*-value < 0.05 was considered statistically significant.

The dataset was randomly divided into a training set (*n* = 312) and a test set (*n* = 135) in a 7:3 ratio. The random sampling method is as follows: Number the samples according to the review time sequence and enter them into an Excel spreadsheet on a computer. Use the formula ‘=RANDBETWEEN (minimum value, maximum value)’ = VLOOKUP(lookup value, data, column index, match condition),” 312 training set sample sequence numbers (70% of the total sample) are randomly generated, while the remaining 135 samples (30% of the total sample) are assigned to the test set. All variables from the training set were initially included in a univariate logistic regression analysis to identify potential predictors. Variables with statistical significance in the univariate analysis were subsequently entered into a stepwise multivariate logistic regression model, using the Akaike Information Criterion (AIC) as the criterion for variable selection. Independent predictors identified in the final multivariate model were incorporated into the construction of a nomogram prediction model.

The predictive performance of the nomogram was evaluated in both the training and test sets using receiver operating characteristic (ROC) curves, calibration curves, decision curve analysis (DCA), and the Hosmer–Lemeshow (HL) goodness-of-fit test. Model discrimination was assessed by calculating the area under the ROC curve (AUC), while calibration was evaluated using calibration plots and the HL test.

Variable coding notes:

1) Gestational diabetes mellitus: Yes = 1; No = 0
2) Gestational hypertension: Yes = 1; No = 0
3) Mode of delivery: Vaginal delivery = 1; Elective caesarean section = 2; Emergency caesarean section = 3
4) Assisted vaginal delivery (forceps): Yes = 1; No = 0
5) Episiotomy: Yes = 1; No = 0
6) Preterm birth: Yes = 1; No = 0
7) Difficult labour: Yes = 1; No = 0

## Results

This study ultimately included 447 subjects aged 19–40 years, with a mean age of 29.1 ± 3.1 years. Including 223 vaginal deliveries, 180 planned cesarean sections, and 44 intrapartum cesarean sections.

### Comparative analysis of general data from training sets and validation sets

The cohort of 447 primiparous women was randomly divided into training and test sets at a 7:3 ratio, yielding 312 cases in the training set and 135 in the test set. No statistically significant differences were observed in clinical characteristics or ultrasound parameters between the sets (*p* > 0.05), confirming their suitability for model development and validation.

### Univariate analysis of clinical data from the training set and various parameters from three-dimensional pelvic floor ultrasound

Based on the presence or absence of SUI, participants in the training set were categorised into the SUI group (*n* = 108) and the non-SUI group (*n* = 204). Given the minimal prevalence of smoking and alcohol consumption (*n* = 1 per group), these variables were not subjected to formal statistical testing as they lacked sufficient power for meaningful analysis. There were statistically significant differences (*p* < 0.05) between the SUI group and the non-SUI group in the training set in terms of mode of delivery, use of assisted delivery techniques (forceps) during delivery, and the presence of episiotomy tears during delivery. There were statistically significant differences between the two groups in the following parameters (*p* < 0.05): urethral tilt angle at rest, bladder neck position during maximum Valsalva manoeuvre, bladder neck mobility, urethral tilt angle during maximum Valsalva manoeuvre, urethral rotation angle, posterior bladder angle at rest, posterior bladder angle during maximum Valsalva manoeuvre, and levator ani muscle hiatus area (Table 1).

**Table 1:** Comparison of baseline characteristics between the two groups of postpartum women in the training set.

Variable	SUI absent (*n* = 204)	SUI present (*n* = 108)	Statistic	*p*-value
Resting retrovesical angle, (°)	116.42 ± 13.53	122.54 ± 13.46	*t* = −3.80	<0.001
Age, (years)	29.00 (27.00, 31.00)	29.50 (27.00, 31.00)	*Z* = –1.02	0.308
Height, (cm)	160.00 (157.00, 164.00)	160.00 (158.00, 165.00)	*Z* =−1.09	0.275
Weight, (kg)	68.00 (62.75, 74.00)	68.00 (63.00, 73.00)	*Z* =−0.06	0.951
BMI, (kg/m^2^)	26.42 (24.61, 28.98)	26.22 (24.38, 28.52)	*Z* =−0.52	0.603
Gestational weight gain, (kg)	13.00 (10.50, 16.00)	13.50 (11.00, 16.25)	*Z* =−0.61	0.544
Gestational age at delivery, (weeks)	39.00 (38.00, 39.00)	39.00 (38.00, 39.00)	*Z* =−0.73	0.463
Birth weight, (kg)	3.28 (2.96, 3.52)	3.22 (2.98, 3.38)	*Z* =−0.86	0.387
Position of the bladder neck at rest, (mm)	26.00 (24.00, 28.00)	25.00 (23.00, 27.00)	*Z* =−1.78	0.075
Bladder neck position under maximum Valsalva manoeuvre, (mm)	14.00 (7.00, 19.00)	−6.00 (−9.00, −2.00)	*Z* =−11.50	<0.001
Bladder neck mobility, (mm)	11.00 (6.00, 18.00)	31.00 (25.00, 36.00)	*Z* =−11.08	<0.001
Resting urethral inclination angle, (°)	30.00 (19.75, 41.00)	23.00 (14.75, 35.00)	*Z* =−3.09	0.002
Urethral inclination angle during maximum Valsalva, M (°)	21.50 (13.00, 35.00)	51.50 (32.75, 65.50)	*Z* =−8.72	<0.001
Urethral rotation angle, M (°)	33.00 (15.00, 55.50)	67.50 (50.00, 87.00)	*Z* =−8.34	<0.001
Retrovesical angle during maximum Valsalva, (°)	128.00 (118.75, 140.00)	149.00 (136.75, 160.00)	*Z* =−7.37	<0.001
Levator hiatus area, (cm^2^)	15.00 (13.00, 18.00)	22.00 (18.00, 25.00)	*Z* =−10.42	<0.001
Neonatal sex, *n* (%)			*χ*^2^ = 3.17	0.075
Male	107 (52.45)	68 (62.96)		
Female	97 (47.55)	40 (37.04)		
Gestational diabetes mellitus, *n* (%)			*χ*^2^ = 0.19	0.667
Absent	186 (91.18)	100 (92.59)		
Present	18 (8.82)	8 (7.41)		
Pregnancy-induced hypertension, *n* (%)			*χ*^2^ = 0.90	0.342
Absent	197 (96.57)	101 (93.52)		
Present	7 (3.43)	7 (6.48)		
Mode of delivery, *n* (%)			*χ*^2^ = 54.68	<0.001
Vaginal deliveries	72 (35.29)	85 (78.70)		
Planned cesarean deliveries	106 (51.96)	15 (13.89)		
Intrapartum cesarean deliveries	26 (12.75)	8 (7.41)		
Use of assisted delivery techniques during labor (forceps), *n* (%)			*χ*^2^ = 19.36	<0.001
Absent	203 (99.51)	95 (87.96)		
Present	1 (0.49)	13 (12.04)		
Episiotomy, *n* (%)			*χ*^2^ = 68.05	<0.001
Absent	182 (89.22)	49 (45.37)		
Present	22 (10.78)	59 (54.63)		
Perineal lacerations, *n* (%)			*χ*^2^ = 2.74	0.098
Absent	157 (76.96)	73 (67.59)		
Present	47 (23.04)	35 (32.41)		
Precipitous labor, *n* (%)			*χ*^2^ = 0.32	0.574
Absent	203 (99.51)	106 (98.15)		
Present	1 (0.49)	2 (1.85)		
Dystocia, *n* (%)			*χ*^2^ = 0.62	0.432
Absent	175 (85.78)	89 (82.41)		
Present	29 (14.22)	19 (17.59)		

Normally distributed continuous variables: mean ± standard deviation (SD); Non-normally distributed continuous variables: median (interquartile range, IQR); Categorical variables: *n* (%).

### Analysis of risk factors for postpartum SUI

Variables demonstrating statistical significance (*p* < 0.05) in univariate analyses were entered into a stepwise multivariable logistic regression model with the Akaike Information Criterion (AIC) as the variable selection criterion to derive the final predictive model. The results showed that the use of assisted delivery techniques (forceps), bladder neck position during maximum Valsalva manoeuvre, urethral rotation angle, posterior bladder angle during maximum Valsalva manoeuvre, and levator ani muscle tear area were risk factors for early postpartum SUI (OR = 34.57, 0.81, 0.98, 1.04, 1.29, respectively, all *p* < 0.05; Table 2).

**Table 2:** Results of multi-factor logistic regression of a nomogram prediction model.

Variable	*β*	SE	Wald *Z*	*p*-value	Adjusted OR (95% CI)
Intercept	−7.99	2.20	−3.64	<0.001	0.00 (0.00–0.02)
Use of assisted delivery techniques during labor (forceps)					
Absent					1.00 (reference)
Present	3.54	1.26	2.81	0.005*	34.57 (2.92 –409.02)
Bladder neck position under maximum Valsalva manoeuvre	−0.21	0.07	−3.21	0.001*	0.81 (0.71–0.92)
Bladder neck mobility	−0.09	0.06	−1.45	0.148	0.92 (0.81–1.03)
Urethral rotation angle	−0.02	0.01	−1.99	0.047*	0.98 (0.97–0.99)
Retrovesical angle during maximum Valsalva	0.04	0.01	4.00	<0.001*	1.04 (1.02–1.07)
Levator hiatus area	0.25	0.06	4.57	<0.001*	1.29 (1.16–1.44)

*β*, regression coefficient; SE, standard error; OR, odds ratio; CI, confidence interval.

^*^Significant values.

### Development and evaluation of a nomogram prediction model

**Figure 2:**
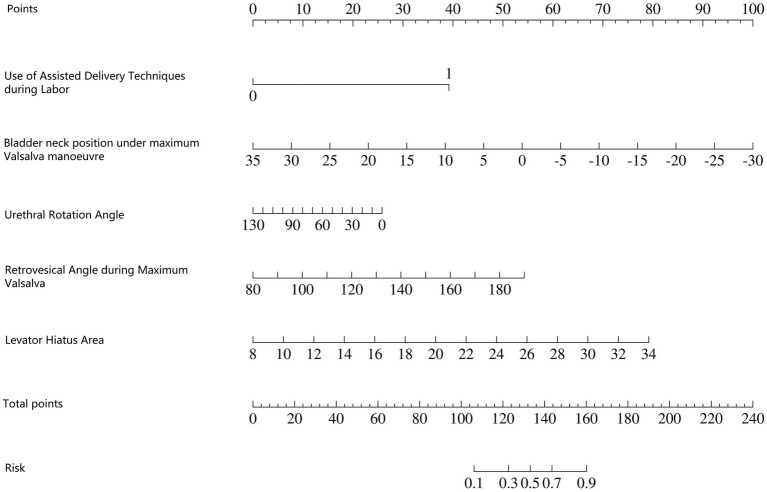
Nomogram model for predicting early postpartum anterior pelvic floor dysfunction.

**Figure 3:**
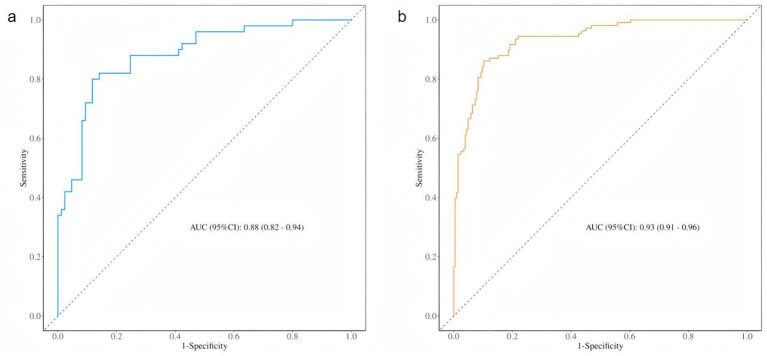
ROC curve of the training set and test set. **(a)** The ROC curve of the training set. **(b)** The ROC curve of the training set.

The independent risk factors identified through stepwise logistic regression-namely, operative vaginal delivery (forceps), bladder neck position during maximal Valsalva, urethral rotation angle, retrovesical angle during maximal Valsalva, and levator hiatus area-were incorporated into a predictive nomogram model (Figure 2). Receiver operating characteristic (ROC) analysis demonstrated excellent model performance, with an area under the curve (AUC) of 0.933 (95% CI: 0.906–0.961) in the training set, indicating a high level of discriminatory ability (Figure 3).

**Figure 4:**
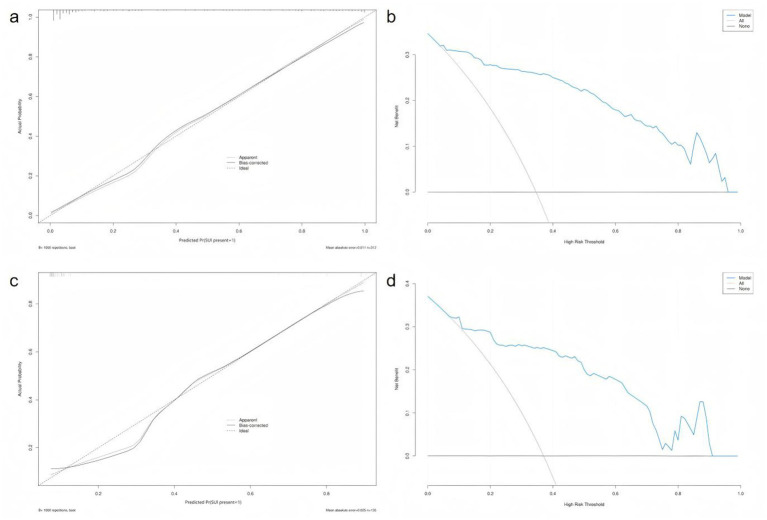
Calibration and decision curve analysis across training and test sets. **(a)** Calibration curve and Hosmer–Lemeshow test of the training set. **(b)** Decision curve analysis (DCA) of the training set. **(c)** Calibration curve and Hosmer–Lemeshow test of the test set. **(d)** Decision curve analysis (DCA) for the test set.

The Hosmer–Lemeshow (HL) goodness-of-fit test for the training set calibration curve yielded a *χ*^2^ value of 10.281 (df = 8, *p* = 0.2458), suggesting good model calibration (as *p* > 0.05). Similarly, the HL test for the test set showed a *χ^2^* value of 7.456 (df = 8, *p* = 0.4884), further confirming the model’s calibration and stability when applied to an independent sample (Figure 4).

The ROC curve of the test set also indicated strong predictive performance, with a high AUC value demonstrating good generalisability. In both the training and test sets, the blue prediction curve consistently outperformed the “None” strategy (black curve) across most threshold levels, indicating that the model performs substantially better than random or uninformative prediction. Furthermore, the model’s performance surpassed that of the “All” strategy, underscoring its effectiveness in identifying individuals at high risk of postpartum SUI.

#### Classic cases

How to use the nomogram model: Based on the patient’s clinical data and pelvic floor ultrasound examination results, scores corresponding to each predictive indicator can be obtained. The sum of the scores for each indicator is the total score. Find the corresponding risk value on the total score line to determine the probability of SUI occurring in the postpartum woman.

**Figure 5:**
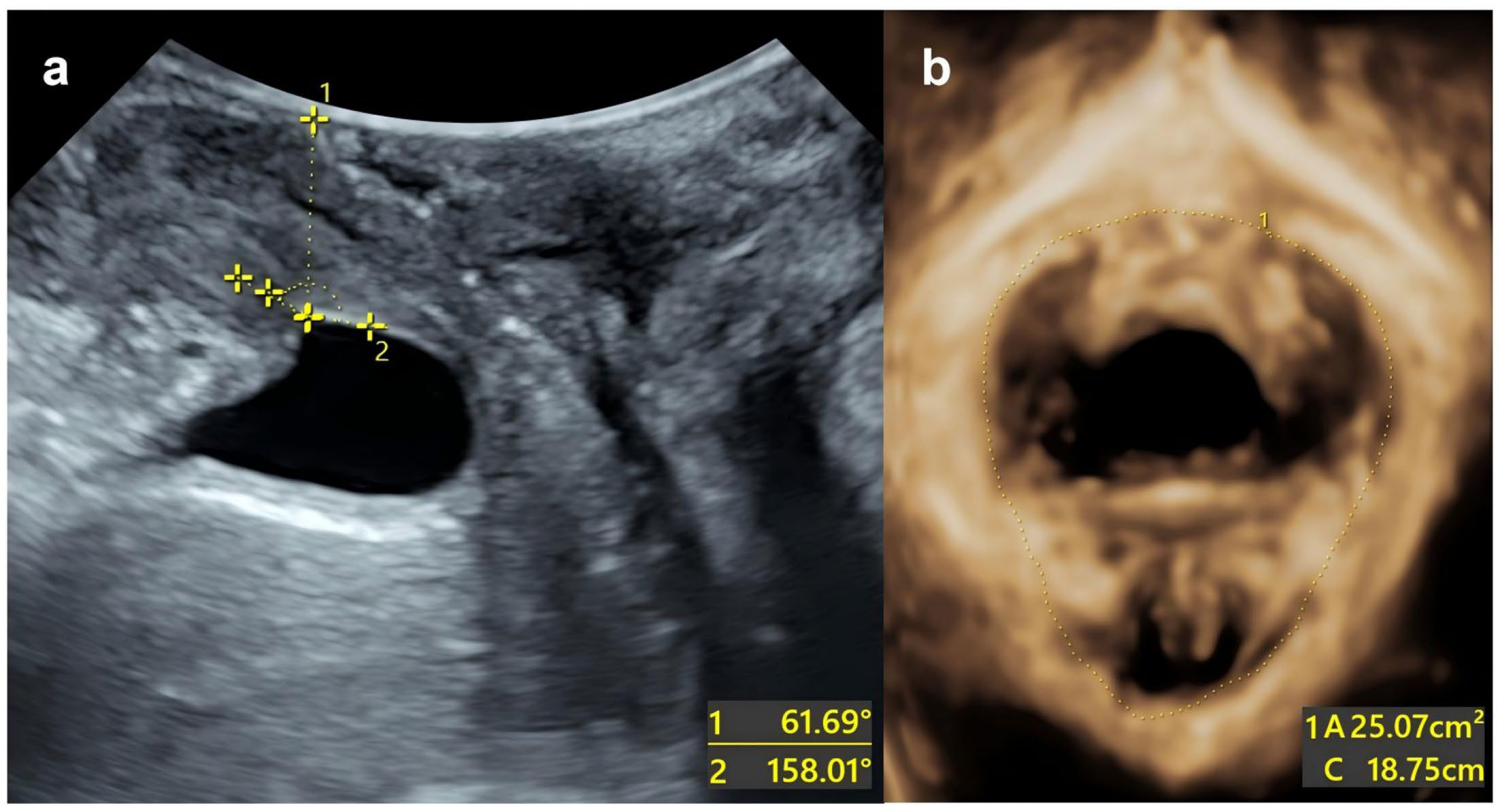
Split-screen image for genital hiatus at the maximum Valsalva maneuver. **(a)** The urethral inclination angle under maximum Valsalva manoeuvre is 61.69°. The posterior corner of the bladder under the maximum Valsalva manoeuvre is 158.01°. **(b)** The area of the levator ani muscle defect is 25.07 cm^2^.

Example: The patient is a 27-year-old primipara who delivered vaginally with forceps-assisted delivery during labour. During the maximum Valsalva manoeuvre, the following measurements were taken: bladder neck position −3 mm; urethral rotation angle 92.8°; posterior bladder angle 158.01°; and levator ani muscle hiatus area 25.07 cm^2^. The total score was 220, which corresponds to a predicted risk of postpartum SUI of approximately >90% (Figure 5).

## Discussion

SUI is the most prevalent subtype of postpartum PFD in women (14), significantly impairing physiological function as well as social and psychological wellbeing. However, the underlying pathophysiology of SUI remains incompletely understood. Current evidence suggests that weakened urethral sphincter function and compromised urethral support structures are the primary mechanisms, contributing to involuntary urine leakage during increased intra-abdominal pressure (15). Early identification of SUI risk is essential for implementing personalised treatment strategies aligned with the principle of “prevention-first, prevention and treatment combined” (16). Nonetheless, most existing predictive studies are retrospective and single-factor in nature, often yielding biased results despite identifying cut-off thresholds.

In this study, we integrated clinical risk factors and three-dimensional pelvic floor ultrasound dynamic parameters into a multivariable analysis framework to construct a clinically applicable and visual nomogram model. This approach facilitates risk stratification and supports early and targeted interventions for postpartum SUI.

Univariate analysis identified significant associations between postpartum SUI and several obstetric factors, including mode of delivery, use of assisted delivery (forceps), and presence of episiotomy or perineal laceration (all *p* < 0.05). Notably, multivariate logistic regression confirmed forceps-assisted vaginal delivery as an independent risk factor (OR = 34.57; 95% CI: 2.92–409.02), consistent with previous findings. Mechanistically, this can be attributed to: (1) the mechanical impact of the fetal head on the levator ani complex, which may stretch pelvic tissues up to 3.3 times their pre-pregnancy length (17); (2) increased intra-abdominal pressure during the second stage of labour, combined with fetal descent, leading to trauma of the pelvic soft tissues, fascia, muscles, and nerves; and (3) direct damage to pelvic floor structures during episiotomy or operative delivery, particularly with forceps assistance, which compromises the integrity of the anterior pelvic compartment and predisposes to SUI (18). A recent study of 92 primiparous women found that levator ani muscle injuries were significantly increased during vaginal delivery (19). Although elective caesarean section may mitigate pelvic floor trauma and reduce SUI risk, it is associated with significantly higher rates of serious complications-such as cardiac arrest, infection, and haematoma-compared to vaginal delivery (20). These findings underscore the importance of stratified clinical decision-making that balances maternal pelvic floor protection with surgical safety.

Three-dimensional pelvic floor ultrasound has become a standard modality for evaluating SUI due to its accessibility, reproducibility, cost-effectiveness, and capacity to provide real-time, dynamic visualization of pelvic structures, particularly in the postpartum screening of primiparas. In the present study, several dynamic ultrasound parameters assessed during maximal Valsalva manoeuvre-including bladder neck position, urethral rotation angle, posterior bladder angle, and levator hiatus area-were identified as independent predictors of postpartum SUI. Notably, an increased anal sphincter tear area was significantly associated with a higher risk of SUI (OR = 1.29; 95% CI: 1.16–1.44), highlighting the importance of pelvic floor muscle integrity. Previous studies have demonstrated that enlargement of the levator hiatus is indicative of levator ani muscle injury, which compromises the structural support of the pelvic floor (21). The levator ani muscle is the principal supportive structure of the pelvic floor; increased muscle thickness is associated with a smaller hiatus and enhanced pelvic support. However, excessive stretching or avulsion during childbirth may lead to compensatory enlargement of the levator hiatus, reduced fascial tension, and diminished pelvic support (22). Consequently, a larger tear area correlates with an increased risk of stress incontinence during episodes of elevated intra-abdominal pressure.

Additionally, bladder neck descent and an enlarged posterior bladder angle during Valsalva were also shown to be independent predictors of postpartum SUI, consistent with established pathophysiological theories (23). According to the pressure transmission theory, sudden increases in intra-abdominal pressure may cause the bladder neck to descend below the pubococcygeal line, altering the posterior bladder angle and impairing the function of the proximal urethral sphincter. This results in the failure of urethral pressure to counterbalance intravesical pressure, thereby leading to incontinence (24). The study by Turkoglu et al. (25) also confirmed that BND has the highest sensitivity and specificity among the parameters for assessing SUI. Furthermore, in this study, a wider posterior bladder angle was significantly associated with an enlarged levator hiatus, suggesting a common pathophysiological mechanism in which weakened pelvic support structures contribute to structural deformation of the posterior bladder wall and vaginal fascia.

Our findings also align with those of Liu et al. (26), who developed a postpartum SUI prediction model based on pelvic floor ultrasound parameters. Their study identified bladder neck mobility and internal urethral orifice funneling as key predictive features. Similarly, our model emphasizes the importance of urethral dynamic parameters-including bladder neck position, rotation angle, and posterior bladder angle-as essential components in the early identification of women at risk for SUI. These characteristics are of particular importance in the clinical assessment of postpartum primiparas.

Nomogram models are increasingly recognized as valuable tools in gynecological risk prediction, offering the advantage of integrating multiple risk factors into a user-friendly, visualized scoring system. These models enable clinicians to quantify individualized risk through intuitive point-based calculations (27). The nomogram developed in this study innovatively incorporates both clinical variables (such as forceps-assisted delivery) and dynamic three-dimensional pelvic floor ultrasound parameters (bladder neck position, urethral rotation angle, and levator hiatus area during Valsalva). Compared with traditional models, our nomogram demonstrated superior diagnostic performance, with an area under the ROC curve (AUC) of 0.933, and provides a clinically feasible and cost-effective strategy for early risk stratification and targeted intervention in postpartum SUI.

Despite these promising results, our study has several limitations. Firstly, this study employed a retrospective design. Although urinary incontinence history was strictly determined based on diagnostic records in medical files, some symptomatic cases may not have been reported to clinicians or documented in detail. This may introduce a degree of selection bias into the results. Future research should adopt a prospective design and utilize validated standardized questionnaires at baseline to assess urinary incontinence status more accurately and comprehensively. Secondly, all data in this study originated from a single center, lacking external validation and thus limiting the generalizability of findings. Future research should conduct large-scale, multicentre studies to validate model efficacy. Thirdly, data collection was limited to the 6–8 weeks postpartum period, without long-term follow-up to assess the persistence or resolution of SUI symptoms over time. Furthermore, the potential effects of perinatal interventions-such as pelvic floor rehabilitation exercises-were not evaluated and may have influenced outcomes. Future research should involve multi-centre, prospective external validation cohorts to refine the model, incorporate additional clinical biomarkers or imaging parameters, and explore the integration of machine learning techniques to enhance the analysis of ultrasound features. Such advancements may further improve the predictive accuracy and clinical utility of postpartum SUI risk assessment models.

## Conclusion

In summary, the nomogram model developed in this study, which integrates clinical risk factors with three-dimensional pelvic floor ultrasound dynamic parameters, demonstrates promising potential as a tool for predicting the risk of postpartum stress urinary incontinence in primiparous women. By facilitating early identification of individuals at higher risk, this model may contribute to improving clinical screening and guiding personalized interventions. Further validation in larger, multicentre cohorts incorporating additional relevant factors is warranted to confirm its generalizability and clinical utility.

## Data Availability

The raw data supporting the conclusions of this article will be made available by the authors, without undue reservation.

## References

[1] Yavuz M Etiler N. Addressing urinary incontinence by gender: a nationwide population-based study in Turkiye. BMC Urol (2023) 23:205. doi: 10.1186/s12894-023-01388-2, PMID: 38071293 PMC10710702

[2] Stafne SN Dalbye R Kristiansen OM Hjelle YE Salvesen KÅ Mørkved S . Antenatal pelvic floor muscle training and urinary incontinence: a randomized controlled 7-year follow-up study. Int Urogynecol J. (2022) 33:1557–65. doi: 10.1007/s00192-021-05028-x, PMID: 34936023 PMC9206614

[3] Jundt K Peschers U Kentenich H. The investigation and treatment of female pelvic floor dysfunction. Dtsch Arztebl Int. (2015) 112:564–74. doi: 10.3238/arztebl.2015.0564, PMID: 26356560 PMC4570968

[4] Wu X Yi X Zheng X Chen Z Liu J Dai X. Knowledge, attitudes, and practice of pelvic floor dysfunction and pelvic floor ultrasound among women of childbearing age in Sichuan, China. Front Public Health. (2023) 11:1160733. doi: 10.3389/fpubh.2023.1160733, PMID: 37234767 PMC10206020

[5] Gao L Zhang D Wang S Jia Y Wang H Sun X . Effect of the app-based video guidance on prenatal pelvic floor muscle training combined with global postural re-education for stress urinary incontinence prevention: a protocol for a multicenter, randomized controlled trial. Int J Environ Res Public Health. (2021) 18:12929. doi: 10.3390/ijerph182412929, PMID: 34948546 PMC8700899

[6] Batmani S Jalali R Mohammadi M Bokaee S. Prevalence and factors related to urinary incontinence in older adults women worldwide: a comprehensive systematic review and meta-analysis of observational studies. BMC Geriatr. (2021) 21:212. doi: 10.1186/s12877-021-02135-8, PMID: 33781236 PMC8008630

[7] Yount SM Fay RA Kissler KJ. Prenatal and postpartum experience, knowledge and engagement with Kegels: a longitudinal, prospective, multisite study. J Womens Health. (2021) 30:891–901. doi: 10.1089/jwh.2019.8185, PMID: 32931374 PMC8336225

[8] Nambiar AK Arlandis S Bø K Cobussen-Boekhorst H Costantini E de Heide M . European Association of Urology guidelines on the diagnosis and Management of Female non-neurogenic Lower Urinary Tract Symptoms. Part 1: diagnostics, overactive bladder, stress urinary incontinence, and mixed urinary incontinence. Eur Urol. (2022) 82:49–59. doi: 10.1016/j.eururo.2022.01.045, PMID: 35216856

[9] Moris L Heesakkers J Nitti V O’Connell HE Peyronnet B Serati M . Prevalence, diagnosis, and management of stress urinary incontinence in women: a collaborative review. Eur Urol. (2025) 87:292–301. doi: 10.1016/j.eururo.2024.12.017, PMID: 39848866

[10] McKinney J Keyser L Clinton S Pagliano C. ACOG committee opinion no. 736: optimizing postpartum care. Obstet Gynecol. (2018) 132:784–5. doi: 10.1097/AOG.0000000000002849, PMID: 30134408

[11] ACOG Practice Bulletin No. 203 Summary. Chronic hypertension in pregnancy. Obstet Gynecol. (2019) 133:215–9. doi: 10.1097/AOG.0000000000003021 30575669

[12] Schwarz P. IDF global clinical practice recommendations for managing type 2 diabetes −2025. Diabetes Res Clin Pract. (2025) 222:112158. doi: 10.1016/j.diabres.2025.112158, PMID: 40204550

[13] Xu XF Chinese Maternal and Child Health Association Midwife Branch. Clinical practice guideline of normal birth. Chin J Perinat Med. (2020) 23:371–5. doi: 10.3760/cma.j.cn113903-20200526-00492

[14] Xue K Palmer MH Zhou F. Prevalence and associated factors of urinary incontinence in women living in China: a literature review. BMC Urol. (2020) 20:159. doi: 10.1186/s12894-020-00735-x, PMID: 33054777 PMC7559450

[15] Yang X Wang X Gao Z Li L Lin H Wang H . The anatomical pathogenesis of stress urinary incontinence in women. Medicina (Kaunas). (2022) 59:5. doi: 10.3390/medicina59010005, PMID: 36676629 PMC9865065

[16] Chen JY Xu JY Huang ZP. Application progress of ultrasound in diagnosis and efficacy evaluation of female stress urinary incontinence. Chin J Endosc Urol. (2025) 19:14–20. doi: 10.3877/cma.j.issn.1674-3253.2025.01.003

[17] Hadad S Oberman M Ben-Arie A Sacagiu M Vaisbuch E Levy R. Intrapartum ultrasound at the initiation of the active second stage of labor predicts spontaneous vaginal delivery. Am J Obstet Gynecol MFM. (2021) 3:100249. doi: 10.1016/j.ajogmf.2020.100249, PMID: 33451615

[18] Chen L Luo D Yu X Jin M Cai W. Predicting stress urinary incontinence during pregnancy: combination of pelvic floor ultrasound parameters and clinical factors. Acta Obstet Gynecol Scand. (2018) 97:966–75. doi: 10.1111/aogs.13368, PMID: 29754393

[19] Cai S Xia M Ding Y Zeng L. Clinical value of transperineal ultrasound in evaluating the effects of different delivery methods on the primipara pelvic floor structure and function. Sci Rep. (2024) 14:23980–7. doi: 10.1038/s41598-024-75014-y, PMID: 39402151 PMC11473786

[20] Liu YY Wang YQ. Research progress on influencing factors of intrapartum conversion to cesarean section under the new labor standard. Int J Gynaecol Obstet. (2021) 48:481–5. doi: 10.12280/gjfckx.20210171

[21] Peker H Haliloglu Peker B. 3D high frequency endovaginal ultrasound evaluation of urethral and pelvic morphology in stress urinary incontinence in first pregnancy. Eur J Obstet Gynecol Reprod Biol. (2021) 261:148–53. doi: 10.1016/j.ejogrb.2021.04.037, PMID: 33940425

[22] Zheng X Zhao Y. Research status of pelvic floor dysfunction during pregnancy and postpartum and its prevention. Chin J Obstet Gynecol Pediatr. (2022) 18:366–72. doi: 10.3877/cma.j.issn.1673-5250.2022.03.017

[23] Zhu X Gao YD Xu C Tang W Xie L Ge Q . Influence of parity and delivery mode on the area of levator hiatus assessed by pelvic floor three-dimensional ultrasound. Chin J Med Ultrasound (Electron Ed). (2022) 19:920–5. doi: 10.3877/cma.j.issn.1672-6448.2022.09.009

[24] Li Q Lei L Zhang X Zhang X Wei SL Duan SL . Establishment of a pelvic floor ultrasonic scoring model for postpartum stress urinary incontinence based on Logistic regression analysis and its diagnostic value. J Clin Ultrasound Med. (2021) 23:836–40. doi: 10.3969/j.issn.1008-6978.2021.11.009

[25] Turkoglu A Coskun ADE Arinkan SA Vural F. The role of transperineal ultrasound in the evaluation of stress urinary incontinence cases. Int Braz J Urol. (2022) 48:70–7. doi: 10.1590/S1677-5538.IBJU.2020.1100, PMID: 34528775 PMC8691236

[26] Xu C Guo Y Chi X Chen Y Chu L Chen X. Establishment and validation of a simple nomogram for predicting early postpartum stress urinary incontinence among women with vaginal delivery: a retrospective study. BMC Womens Health. (2023) 23:8. doi: 10.1186/s12905-023-02160-2, PMID: 36624424 PMC9827703

[27] Li NX Mai XX Zhang QZ Zhang Y Dou DY Chen YQ . Clinical value of pelvic floor four-dimensional ultrasound combined with pelvic floor surface electromyography in evaluating bladder prolapse in primiparous women with different delivery methods. Chin J Ultrasonogr. (2024) 33:427–33. doi: 10.3760/cma.j.cn131148-20231224-00314

